# The Structure Stability of Metal Diffusion Membrane-Filters in the Processes of Hydrogen Absorption/Desorption

**DOI:** 10.3390/membranes12111132

**Published:** 2022-11-11

**Authors:** Olga V. Akimova, Roman D. Svetogorov, Alexey V. Ovcharov, Nataliya R. Roshan

**Affiliations:** 1 Physics Department, Lomonosov Moscow State University, 119991 Moscow, Russia; 2 National Research Centre “Kurchatov Institute”, 123182 Moscow, Russia; 3 Baikov Institute of Metallurgy and Materials Science, 119334 Moscow, Russia

**Keywords:** X-ray diffraction, palladium—lead membrane alloys, SEM-EDXS analysis, hydrogen

## Abstract

The evolution of a nanostructured state of palladium—lead membrane alloys during their interaction with hydrogen was studied using precision X-ray diffraction with synchrotron radiation (SR) and scanning electron microscopy (SEM) with energy-dispersive X-ray spectroscopy (EDXS). The importance of this topic is due to the need and demand for improving the performance characteristics of dense metal diffusion filters for high purity hydrogen separation processes. Palladium-based membrane filters with lead concentrations of 5 and 20 wt.% were prepared via electric arc melting from high purity metals (99.95%). The thickness of the filters was 50 μm. Hydrogenation was carried out from a gas medium at 573 K and the pressure of 16 atm. within 150 min. The focus of the study is on the structural state of diffusion filter membranes depending on the content of the palladium-alloying element—lead—and on analysis of the substructure of alloys before and 5300 h relaxation after hydrogenation is carried out. Specific features of the surface morphology and the structure of the membrane filters depending on the concentration of lead in the alloys are determined. The formation and development of deformation processes in metal systems upon the hydrogenation is shown. The establishment of peculiarities of hydrogen interaction with metals will contribute to obtaining new potentially important characteristics of membrane filters.

## 1. Introduction

The separation of high-purity hydrogen from hydrogen-containing gas mixtures using the diffusion method via using dense metal membranes of palladium alloys is an important direction of hydrogen energy research [1,2,3,4]. The palladium-based membrane alloys inherit their unique property of selective permeability to hydrogen; therefore, they remain the leaders of diffusion methods for obtaining high-purity hydrogen [2,3,4,5]. Alloying elements are added to palladium in order to improve the strength characteristics and hydrogen permeability of diffusion membrane filters [2,3,4,6,7]. The enhancement of alloy compositions is carried out not only to achieve improved the performance of membrane filters, but also to increase the reliability and durability of their working condition [7,8,9,10,11].

Local dilatations of the crystal lattice, arising due to the need of palladium doping with various elements, causes imperfections in the structure of membrane filters, which in the processes of hydrogen absorption/desorption can lead to the generation of microcracks [4,8,11,12]. The appearance and development of microcracks affect both the degree of purity of the hydrogen extracted from the gas mixture and the durability and safety of the membrane metal filters. Hydrogen insertion into the metal’s crystal lattice, including but not limited to palladium-based alloys, leads to a strong plastic deformation in the hydrogenated structures [4,7,13]. The problems are caused by phase transformations in hydrogenated materials, (α ↔ β): α-weak solution of hydrogen in the alloy (0.2–0.3 parts of H) and β-hydride in the alloy (upon 0.6–0.8 parts of H) [11,13].

In a number of palladium-based membrane alloys being developed to solve the economic and environmental problems of hydrogen energetics and the automotive industry [1,2,3,4,6,7], palladium alloys with lead are new and insufficiently studied. Our study of them in the processes of hydrogen absorption/desorption is motivated by the fact that doping palladium with lead increases the strength of diffusion membranes, does not reduce their plasticity, increases specific hydrogen permeability, and reduces the temperature of the α ↔ β transition [6]. The ability of membranes to operate at temperatures for diffusion separation of high-purity hydrogen (573–873 K [3,6,7,8]) in the absence of transformations α ↔ β reduces the probability of internal intense plastic deformations of metal systems, which promotes the development of microcracks in the field of interactions with explosive hydrogen.

In our previous work on palladium—lead alloys during hydrogenation at the pressure of 16 atm. at 573 K [14,15,16], a number of significant results were obtained: (i)—it was determined that only the hydride phase is formed in the samples [14]; (ii)—it was found that the membranes have a highly dispersed substructure before hydrogenation [14,15]; (iii)—they restore the initial (before hydrogenation) characteristics of the structural state quite well and do not show a tendency of surface flecking, which is characterization of the palladium—yttrium alloy systems [11]. However, we revealed the predisposition of alloys of this system to the formation of funnel-shaped ulceration in the surface, which is an undesirable effect of the interaction with hydrogen.

The aim of this work is to study in-depth the processes of absorption/desorption of hydrogen by membranes of palladium alloys with lead contents of 5 and 20 wt.% (denoted in the text as membranes *1* and *2*, respectively), as well as to consider the stability of their substructure after hydrogenation and prolonged (5300 h) relaxation.

## 2. Samples and Experiment

The samples were rolled-up plates with a thickness of 50 μm. These plates were created by cold rolling with intermediate vacuum annealing at 1223 K from blanks obtained by electric arc melting in a protective helium atmosphere [6]. For the manufacture of membranes, metals with a purity of 99.95 wt.% were used. The phase composition, microstructure, properties, and mechanism of hydrogen accumulation for membrane filters are investigated using X-ray and electron diffraction methods.

Diffraction spectra in the angular range 1–60° were extracted from the membranes using X-ray diffraction on high-precision equipment of the Kurchatov Research Center [17]. The SR beam was directed at the samples. This was monochromatized using a two-crystal silicon monochromator with the main reflection from the {111} type planes up to ΔE/E~10^−4^. During the measurement, the samples were rotated around a horizontal axis perpendicular to the SR beam for averaging over the orientations of the crystallites. The LaB_6_ standard is used for obtaining accurate lattice parameters. The diffraction patterns were recorded in the Debye—Scherer geometry. The experimental X-ray spectra were processed using the software “Dionis” and Fityk-0.9.8 [18,19]. The method of approximations was used in the analysis of diffraction maxima [20].

Hydrogenation of the samples was carried out once with hydrogen gas on a vacuum installation of the Sieverts type for 573 K and pressure 16 atm. The samples were cooled together with the furnace. The hydrogen content in the crystal lattice of the membranes after hydrogenation was determined by changing its constant [13]. The relaxation of diffusion filter-membranes took place at room temperature and external atmospheric pressure. The calculation of the content of the vacancies in non-hydrogenated alloys is carried out according to Formula (1) [21]:

(1) nvnM=3·(Δa)0.22·a

where ∆*a* is the change in the period of the crystal lattice as a result of the introduction of vacancies, *a* is the period of the crystal lattice of the main phase of the alloy, and 0.22 is the change in the volume of the unit cell when the vacancy is introduced. Taking into account the energy advantage related to the formation of hydrogen vacancy clusters [22], the coefficient of change in the volume of a unit cell after hydrogenation was assumed to be 0.36.

The microstrains in crystallites were evaluated using the Stokes—Wilson (S—W) and Williamson—Hall (W—H) methods [23,24].

The surface photographs of the membranes before and after hydrogenation were studied in backscattered and secondary electrons using a scanning electron microscope Supra_MSU and a dual beam scanning electron microscope Helios NanoLab 600i, equipped with an EDXS system. The microanalyses by EDXS were performed at 4–20 kV. The images of the surface were obtained in high resolution at the nanoscale. The grain boundaries were visible without etching. The EDXS method is insensitive to hydrogen but showed the distribution of the main elements of the membrane alloy when it was in a hydrogenated state. The relative accuracy of the concentration determination was estimated as ±0.1%.

According to the Kanaya—Okayama formula [25], the signal generation depth (r) was 1.3 μm in our samples:

(2) $$r=\frac{0.0276\cdot A\cdot E^{1.67}}{\rho \cdot Z^{0.89}}$$

where A is the average atomic mass, E is the energy of the excitation electron, ρ is the density, and Z is the average atomic number of the material.

## 3. Results and Discussion

### 3.1. X-ray Diffraction

**Figure 1:**
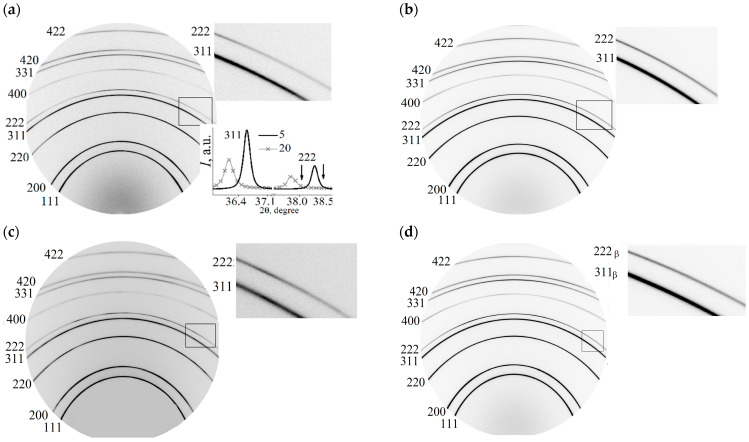

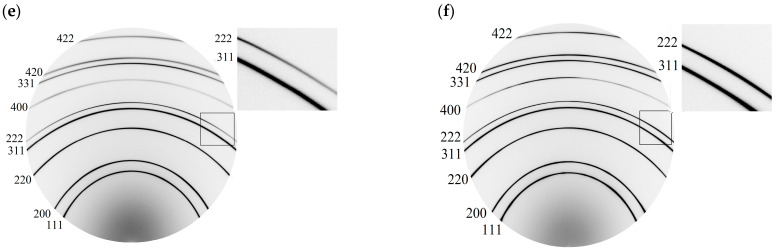
Diffractograms of membranes with a lead content of 5 and 20 wt.% for the states: (**a**,**b**)—before hydrogenation; (**c**,**d**)—24 h relaxation after hydrogenation; (**e**,**f**)—5300 h relaxation after hydrogenation, respectively.

Diffractograms of membrane alloys *1* and *2* are shown in Figure 1.

The main result that follows from them is that the face-centered unit cell in both alloys persists during hydrogenation and subsequent long-term relaxation after it. There was a change in the intensity of reflections from coherent scattering regions (CSR) with Miller indexes of 311 and 111 (CSR (311) and CSR (111) farther), which reveals a rotation of the blocks of polycrystalline samples, as shown in the side insets in Figure 1. For the Pd—In—Ru alloy, a similar result was observed previously: the weakening of the membrane filter texture as a result of a reversal of polycrystal mosaic blocks after directional (electrolytic) hydrogenation of the membranes [26].

**Figure 2:**
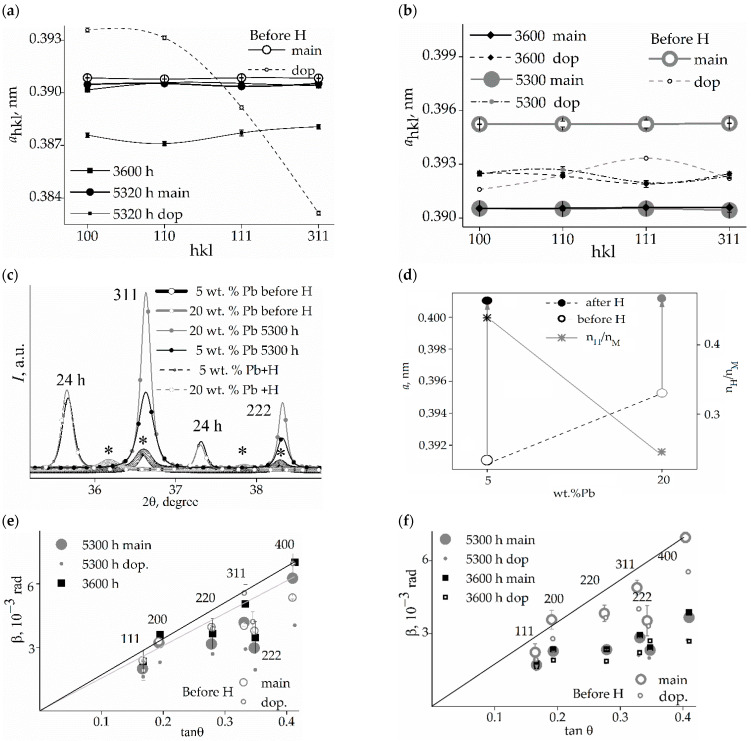
Parameters of elementary cells of the samples for states of relaxation after hydrogenation (before H, 3600 h; 5300 h): (**a**,**b**)—5 and 20 wt.% lead, respectively, (**c**)—the changes in angular position of reflections from CSR (311) и CSR (111) as a result of absorption/desorption processes; (**d**)—the changes in the constant crystal lattice of alloys and the amount of absorbed hydrogen for the state of 24 h relaxation; (**e**,**f**)—the physical broadening of diffraction reflections for the main phase of membranes *1* and *2* by the tanθ.

The bottom Inset in Figure 1a shows the diffraction maxima from the CSR (311) and CSR (111) of both membranes. The arrows indicate the asymmetry of the diffraction peaks at the diffraction angle increases. The asymmetry of diffraction reflections reveals the presence of additional phases of low volume quantities in alloys with partially coherent boundaries and, compared to the main phase of the alloys, with lower unit cell parameters for CSR (311) and CSR (111). The membrane lattice parameters for states before and after hydrogenation (3600 and 5300 h relaxation) are shown in Figure 2 (the error of determination for the main phase is below the size of the symbols). The period of the main phase of membrane *1* before hydrogenation is 0.39054 ± 0.00008 nm, which reveals a lead concentration of 5.03 ± 0.03 wt.% (2.76 ± 0.01 at.%). For membrane *2*, the main phase crystal lattice period is 0.39525 ± 0.00014 nm, which reveals 20.58 ± 0.05 wt.% (11.29 ± 0.05 at.%) of lead.

For membrane *1*, an additional phase with partially coherent boundaries to the main phase was determined to be of 0.05 ± 0.01 vol.%. This phase has an almost isotropic distribution in the CSR (hkl). The larger lattice parameters of the additional phase along the <100> and <110> crystallographic directions, as compared to those along the <111> and <311> crystallographic directions and to parameters of the main phase (Figure 2a), indicates a higher concentration of lead in this phase. In CSR (111) and CSR (311), the additional phase can also have more vacancies, up to 0.10. As is known, a lower energy of vacancy formation leads to a higher concentration of thermodynamically stabilized vacancies in metal systems [27]. Vacancies reduce the charge density near themselves [27,28], so they play an important role in the processes of self-diffusion of atoms of metal systems. It can lead to the development of pores and microcracks [4,28,29], i.e., degradation of strength characteristics and embrittlement of the materials, which is a fundamental problem in the physics of materials.

For membrane *2*, the additional phase is distributed in the lattice anisotropically. Its amount varies from 0.03 (CSR (100)—the minimum) to 0.09 volume fraction—the maximum in CSR (111). The smaller lattice parameter of the additional phase than that of the main phase (Figure 1b) can indicate a depletion of lead or an enrichment in vacancies, whose fraction may be as high as 0.12 in CSR (100), 0.07 in CSR (111), and 0.10 in CSR (311) and in CSR (110). The two-component alloy composition does not allow us to distinguish between these two mechanisms. The accuracy of the X-ray determination of the crystal lattice constant of the main phase of alloys is an order of magnitude higher in comparison with the accuracy of determining the lattice constant of the additional phase of a small fraction.

Diffractograms of type *I*(2θ) (Figure 2c) show a shift of diffraction peaks from CSR (311) and CSR (111) towards a decrease in diffraction angles after hydrogenation (state of 24 h relaxation) compared with similar reflexes obtained for the state of samples before hydrogenation (marked by *). Such a shift indicates an increase in the parameters of the unit cells of alloys as a result of the introduction of hydrogen into the crystal lattice. An increase in the parameters of the crystal cells of the alloys showed that after 24 h of relaxation following hydrogenation in membrane *1* (5 wt.% of lead), the amount of hydrogen is twice as large as that in membrane *2* (20 wt.% of lead) (Figure 2d).

Changes in the intensity of reflections reveal changes in the reflecting volume of crystallites. A more significant change in the reflecting volume is found for membrane *2* (Figure 2c) and it is more substantial when hydrogen leaves the structure, which determines the significant role of vacancies in this alloy.

The physical broadening of the diffraction maxima indicates that after 5300 h of relaxation following hydrogenation, the state of the crystal lattices can be considered as conditionally stable (Figure 2e,f). For this relaxation time, the lattice period of the main phase in the alloy with 5 wt.% lead is 0.390560 ± 0.00001 Å. The lattice period of an additional phase with partially coherent boundaries to the main phase is 0.38850 ± 0.00012 Å. The additional phase is presumably enriched with vacancies up to 0.04. The average content of this phase in CSR (hkl) is 5 vol.%. The elementary cell parameters of the phases (main and additional) for the 5300 h relaxation state show a greater residual effect of hydrogenation for the additional phase than for the main phase in membrane *1* (Figure 2a).

In the case of the alloy with 20 wt.% of lead, more significant changes in the unit cell parameters are revealed for the main phase than for the additional one: the enrichment of vacancies to 0.13 fractions was found, while the unit cell parameters of the additional phase are restored to their values for the state before hydrogenation (Figure 2b).

The predominant influence on the diffraction pattern of microstrains in crystallites of membrane *1* was found: a linear approximation of the physical broadening was obtained; see Figure 2e. The state appearing as the result of hydrogenation begins to form by around 3600 h of relaxation. Comparison of the ratio of tanθ and cosθ (θ is the reflection angle) reveals an increase in the influence of microstrains on the elastically soft crystallographic direction for palladium alloys <100> [20]. For the alloy with 20 wt.% of lead, the picture is dissimilar (Figure 2d); here, the effect of microstrains in the crystallographic direction <100> is visible in the initial (before hydrogenation) state. Hydrogen eliminates this.

**Figure 3:**
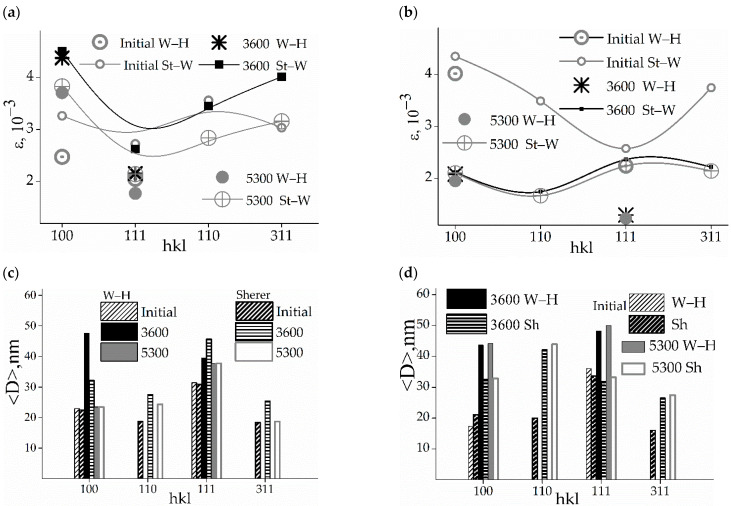
Microstrains in crystallites determined by the Williamson—Hall and the Stokes—Wilson methods [23,24]: (**a**,**b**)—for samples of 5 and 20 wt.% Pb, respectively; and effective sizes <D_hkl_> of CSR (hkl): (**c**,**d**)—for samples of 5 and 20 wt.% Pb, respectively.

The main phase of the alloy with 5 wt.% of lead more accurately restores the characteristics of its initial state than in the case of the lead concentration of 20 wt.% (Figure 3).

After 5300 h of relaxation following hydrogenation, the microstrains in CSR (110), CSR (111), and CSR (311) returned to their values in the initial state of structure or decreased (Figure 3a,b) in both membranes. Some increase is found only for CSR (100) using both methods (S—W and W—H). Specifically, the microstrains in CSR (100) for the state before hydrogenation exceeded those in CSR (111) by 1.2 times (both methods), for the state with 3600 h of relaxation after hydrogenation by 1.7 (S—W) or 2.0 (W—H) times, and by 1.8 (S—W) or 2.0 (W—H) times for the relaxation state with 5300 h following hydrogenation (Figure 3c).

After a single hydrogenation, the alloys recovered their original nanodispersive states (Figure 3c,d). This recovery was more complete in the alloy with 5 wt.% lead. In those with 20 wt.% lead, some anisotropy in the effective sizes of crystallites is more noticeable than in the case of 5 wt.% lead.

### 3.2. SEM and EDXS Analysis Results

**Figure 4:**
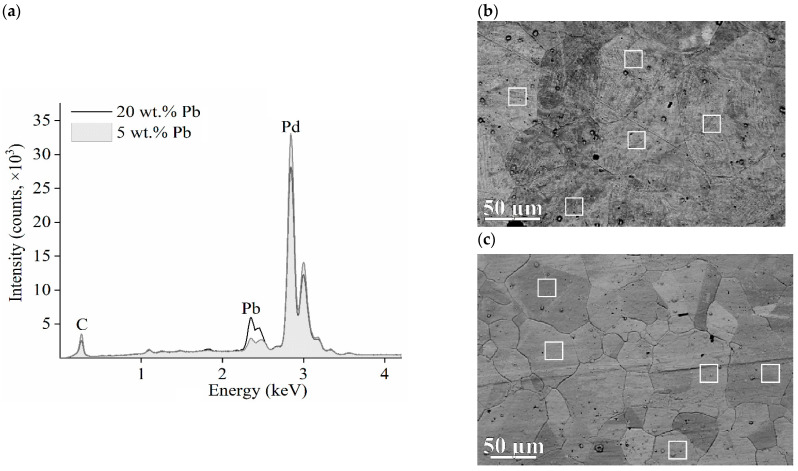
Results of element analysis of Pd/Pb membranes. (**a**)—Typical EDXS spectrums of membranes; SEM images of the surface (**b**)—membrane *1*; (**c**)—membrane *2*. The white frames indicate areas of EDXS analysis.

Figure 4 shows the results of the EDXS analysis. More clear outlines of grain boundaries can be seen in the SEM images of membrane *2*, i.e., for the membrane alloy of 20 wt.% lead (Figure 4c).

Table 1 shows the results of EDXS analysis obtained from samples for five measurement sites for each of the membranes. The sites are marked as white-framed rectangles in Figure 4b,c. For membrane *1*, the stoichiometric ratio of palladium and lead (Table 1), determined using the EDXS method, agrees with the X-ray diffraction results better than in the case of membrane *2*.

**Figure 5:**
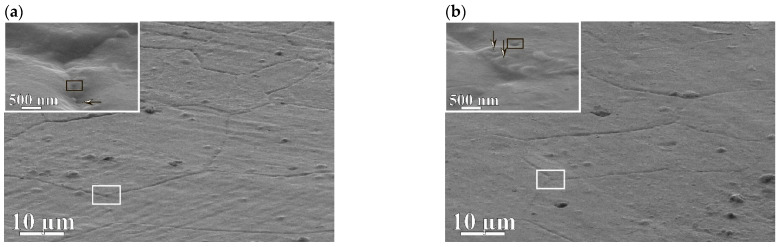

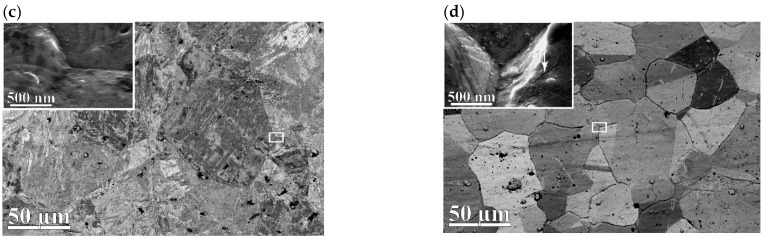
Morphology of the membranes surface: (**a**)—5 wt.% Pb; (**b**)—20 wt.% Pb before hydrogenation; (**c**,**d**)—5 and 20 wt.% Pb, after hydrogenation, respectively. Arrows of the inserts indicate funnel–shaped defects.

Figure 5a,b presents SEM analysis of the surface of the membranes in secondary electrons—the state before hydrogenation. The micrographs were measured at a detector angle of 70° to the membrane surface to obtain more complete information about its features. The images revealed a tightly packed mosaic of grains in the form of polyhedra with almost straight boundaries. The insets show a nanodisperse columnar structure and funnel–shaped defects (marked as rectangles).

Figure 5c,d are backscattered electron images of the surfaces of membranes *1* and *2* after hydrogenation. After interacting with hydrogen, the preservation of higher-angle grain boundaries compared to membrane *1* for membrane *2* was noted (Figure 5d). More frequent cavitation effects on the surface were also detected for membrane *2*. More imperfections in membrane *2* grain boundaries than in those of membrane *1* may be the result of three significant reasons: (i) differences in electronegativity (1.8 for Pb and 2.2 for Pd by Pauling [30]), (ii) differences in melting temperatures of the elements composing the alloys (600 K for Pb and 1825 K for Pd), and (iii) differences in the sizes of Pd and Pb atoms.

The second and third reasons are considered to be the main ones, since if the difference in electronegativity values is about 5%, then the homological temperatures of palladium and lead characterizing the rate of diffusion deformation during the relaxation of alloys after hydrogenation differ by 2.5 times (0.5 for lead vs. 0.2 for palladium), which in percentage terms is 200%. The discrepancy between the crystal lattices of palladium and lead corresponds to 27.2% according to:

(3) $$\Delta =\frac{d_{\mathrm{Pd}}{-d}_{\mathrm{Pb}}}{d_{\mathrm{Pd}}}$$

where *d_Pd_* and *d_Pb_* are structural constants of crystal lattices of palladium and lead (0.3810 and 0.4950 nm, respectively [31]).

So, with an increase in the palladium—lead of the metallic system fraction of lead (membrane *2*), the influence of factors (ii) and (iii) is more significant than in the case of membrane *1*.

## 4. Conclusions

The possibility of obtaining a high degree of homogeneity of the composition of dense diffusion metal filters with lead concentrations of 5 and 20 wt.% is shown.

The structural features of palladium—lead membrane alloys depending on the lead percentage were studied.

Differences in the formation of grain boundaries depending on the amount of lead in the alloys were established.

The influence of lead in membrane alloys on the processes of hydrogen absorption/desorption was established: a membrane filter with lead of 5 wt.% absorbs more hydrogen than that with the lead of 20 wt.%. The alloy of 5 wt.% lead recovered the initial characteristics of the nano-dispersed substructure better after hydrogenation than that with the lead of 20 wt.%.

Based on the obtained results, structural models can be developed that provide the ability to predict properties of membrane alloys and will thus improve the development of new materials with required properties.

## Figures and Tables

**Table 1:** The EDXS analysis results of the composition of membrane filters.

Membranes		wt.%	<wt.%>	at.%	<at.%>
1	Pd	95.6//95.4//95.5//95.7//95.3	95.5	97.7//97.6//97.6//97.7//97.5	97.6
	Pb	4.4//4.6//4.5//4.3//4.7	4.5	2.3//2.4//2.4//2.3//2.5	2.4
2	Pd	86.3/86.2//86.3//86.2//86.8	86.4	92.5//92.4//92.5//92.4//92.7	92.5
	Pb	13.7//13.8//13.7//13.8//13.2	13.6	7.5//7.6//7.5//7.6//7.3	7.5

## Data Availability

Not applicable.
